# Blockade of PI3K/AKT pathway enhances sensitivity of Raji cells to chemotherapy through down-regulation of HSP70

**DOI:** 10.1186/1475-2867-13-48

**Published:** 2013-05-24

**Authors:** Xiaosheng Fang, Yujie Jiang, Lili Feng, Haiping Chen, Changqing Zhen, Mei Ding, Xin Wang

**Affiliations:** 1 Department of Hematology, Provincial Hospital Affiliated to Shandong University, Jinan 250021, P.R. China; 2 Department of Diagnostics, Shandong University School of Medicine, Jinan 250021, P.R. China; 3 Department of Cardiology, Provincial Hospital Affiliated to Shandong University, Jinan 250021, P.R. China

**Keywords:** Raji, Hyperthermia, HSP70, PI3K/AKT

## Abstract

Up-regulation of heat shock protein 70 (HSP70) could be elicited primarily by heat in former studies, and this was proved to be associated with cancer progression. Burkitt's lymphoma is one of highly aggressive B-cell non-Hodgkin’s lymphoma and is one of the fastest growing human tumors. To investigate the effect of HSP70 expression on the sensitivity of human Burkitt lymphoma cells (Raji cells) to chemotherapy and its role in the involvement of PI3K/AKT pathway, we evaluated the effects of LY294002, a PI3K inhibitor, on the expression of HSP70 and cell sensitivity to adriamycin (ADM) or cisplatin (DDP). In present study, expressions of HSP70, AKT and phosphorylated AKT (p-AKT) in Raji cells were measured by Western-Blot. Apoptosis index of Raji cells was examined by flow cytometry. Cytotoxicities of adriamycin (ADM) and cisplatin (DDP) were determined by WST-8 assay. We found that hyperthermia (42 degrees for 1 hour) up-regulated the expression of HSP70 expression and blockade of PI3K/AKT pathway down-regulated HSP70 expression in Raji cells. Compared to cells treated with ADM or DDP alone, hyperthermia protected cells from chemotherapy while LY294002 enhanced sensitivity of Raji cells to chemotherapy. Our results suggested down-regulation of HSP70 expression by blockade of PI3K/AKT pathway maybe responsible for the increased sensitivity of Raji cells to chemotherapy. Targeting PI3K/AKT pathway or inhibiting HSP70 expression may be beneficial for chemotherapy treatment of Burkitt lymphoma patients.

## Introduction

Burkitt lymphoma (BL) is an aggressive, highly proliferative lymphoma, in spite of its low incidence throughout most of the world [1]. It can often be cured by intensive chemotherapy, but the toxicity of such therapy precludes its use in the elderly and in patients with endemic BL in developing countries [2]. Novel therapeutic strategies are required to overcome drug toxicity following conventional treatments.

HSP70 is an important cellular chaperone. Its expression is induced by a wide variety of physiological and environmental insults and allows the cells to survive lethal conditions [3,4]. HSP70 is over-expressed in various human cancers, including pancreatic cancer [5], bladder cancer [6], colon cancer [7], breast cancer [8] and non-Hodgkin’s lymphoma [9]. In cancer cells, over-expression of HSP70 is thought to provide a survival advantage because it is able to interact with multiple components of both the caspase dependent and independent apoptotic pathways [10]. The expression of HSP70 has been routinely associated with cancer progression, therapy responses and poor prognosis in multiple forms of cancers [11,12] and it can be used as a novel biomarker for early detection of cancers. HSP70 also has emerging as a therapy target in cancers recently. Gene silencing of HSP70 inhibits human cancer cell growth in vitro and in vivo [13] and leads to loss of invasion in a variety of cancer cells [14].

Activation of heat shock factor 1(HSF1) is the key step in the induction of HSP70 expression. HSF1 exists as an inactive monomer in a complex with HSP40/HSP70 and HSP90. In such stress as elevated temperature, HSF1 is released from the chaperone complex and transported into the nucleus where it is hyperphosphorylated and binds to DNA containing heat shock elements (NGAAN), leading to rapid induction of these important molecular chaperones [3]. Recently, studies revealed the abolishment of serine473 phosphorylation in Akt could significantly reduce its kinase activity in CML cells. Inactivation of Akt pathway subsequently blocked serine9 phosphorylation of Gsk3β. Active non-phosphorylated Gsk3β rendered HSF1 transcriptionally inactive and reduced HSP70 production. And blocking PI3K/Akt activity also be demonstrated could induce the similar effects on HSP70 expression [15].

Given the role of HSP70 in tumors and the involvement of PI3K/Akt activity in HSP70 expression, it is reasonably plausible that blockade of PI3K/Akt pathway maybe affect HSP70 production and then up-regulate chemotherapy sensitivity in tumor cells. In present study, we demonstrated that hyperthermia treatment (HT) induced HSP70 expression was mediated by PI3K/AKT pathway in Raji cells. Furthermore, blocking of PI3K/AKT pathway could down-regulate HSP70 expression, inhibit cancer cells survival and promote cells apoptosis when co-treated with ADM or DDP. The decrease of HSP70 expression maybe at least partially responsible for the increased chemotherapy sensitivity of Raji cells. Targeting PI3K/AKT pathway may provide new method to improve chemotherapy effects in cancer patients.

## Materials and methods

### Chemicals

LY294002, a PI3K inhibitor (CST,Danvers, MA, USA), was dissolved in 100% dimethyl sulfoxide (DMSO) (Sigma, St. Louis, MO, USA) and stored at -20°C. The stock concentration was 10 mmol/L and the final concentration of DMSO in medium did not exceed 0.05%. At this level, DMSO did not influence cell viability. Adriamycin (ADM) was purchased from Shanghai Pharma, China and cisplatin (DDP) was obtained from Qilu Pharmaceutical co., LTD, Shandong, China. They were dissolved in deionized water to a concentration of 1.0 mg/ml, and then were diluted to defined concentrations for further experiment.

### Cell culture

Raji cells were purchased from Shanghai Institutes for Biological Sciences (SIBS, CAS, Shanghai, China) and maintained in RPMI-1640 medium (Gibco, Carlsbad, CA, USA) containing 10% heat-inactivated fetal bovine serum albumin (Sijiqing Biological Engineering Materials Co., Ltd., Hangzhou, Zhejiang, China), penicillin (100 U/ml), and streptomycin (100 mg/ml) in the 95% humidified incubator with 5% CO_2_ at 37°C. To explore the effects of HT on cell growth and HSP70 expression, Raji cells were seeded on coverslips in 24-well microplates and cultured at 42.0°C for 1 hour, and then immediately returned to 37.0°C for continuous incubation of 2, 4, 8 or 24 hours. To analyze the effects of LY294002 on cell growth and expression of HSP70, Raji cells were cultured with or without LY294002 (5 μM, 10 μM, 20 μM, 40 μM,) for 8 h. For experiments with ADM or DDP treatment, Raji cells were treated with LY294002 or HT (42.0°C for 1 hour) before being exposed to chemicals. Cells were then received continuous incubation of 8 hours at 37.0°C. At last, cells were collected for apoptosis assay and immunoblot analysis of HSP70.

### Flow cytometric analysis

After treatment of Raji cells with LY294002 / HT, or together with ADM or DDP for the indicated periods, Apoptosis index (AI) were analyzed by staining the cells with fluorescein isothiocyanate (FITC)–annexin V and propidium iodide (PI), according to the manufacturer’s instructions (Jingmei Biotech, Shenzhen, China). Briefly, an aliquot of 10^5^ cells was incubated with FITC–annexin V and PI for 15 minutes at room temperature in the dark. Cells were then immediately analyzed by a Beckman Coulter EPICS XL flow cytometer (Beckman Coulter, Fullerton, CA, USA) and variables were analyzed by Expo 32 software.

### Immunoblot analysis

Total protein was extracted from Raji cells in different groups using RIPA and 1% PMSF (CST,Danvers, MA, USA). HSP70 monoclonal antibody was purchased from R&D Systems (Minneapolis, MN, USA). Akt and p-Akt monoclonal antibody were purchased from Cell Signaling Technology (Danvers, MA, USA). The measurement of protein concentrations and detailed procedures of immunoblot analysis were described previously [16].

### Assessment of cell viability

After received LY294002 or hyperthermia treatment as described previously, cells (1 × 10^5^ ) were seeded into 96-well plates. ADM and DDP were then added into cultures. Twenty-four hours later, 10 μL CCK-8 (Dojindo Molecular Technologies, Kumamoto, Japan) was added to each well, and the cells were incubated at atmosphere for 4 hours. The absorbance at 450 nm was then measured using a microplate reader. Percentage of survival cell was calculated as follows: (*A*exp group-Ablank/*A*control-Ablank) × 100%.

### Statistics

Analyses of data were performed by using SPSS15.0 for Windows. Data are presented as the mean ± SD. Differences in the results for two groups were evaluated by Student’s t-test. Half maximal inhibitory concentration (IC50) was analyzed with the linear regression. All experiments were repeated at least three times. All differences were considered to be statistically significant when the *P* value was less than 0.05.

## Results

### Effects of HT and LY294002 on cell apoptosis and expression of HSP70

**Figure 1:**
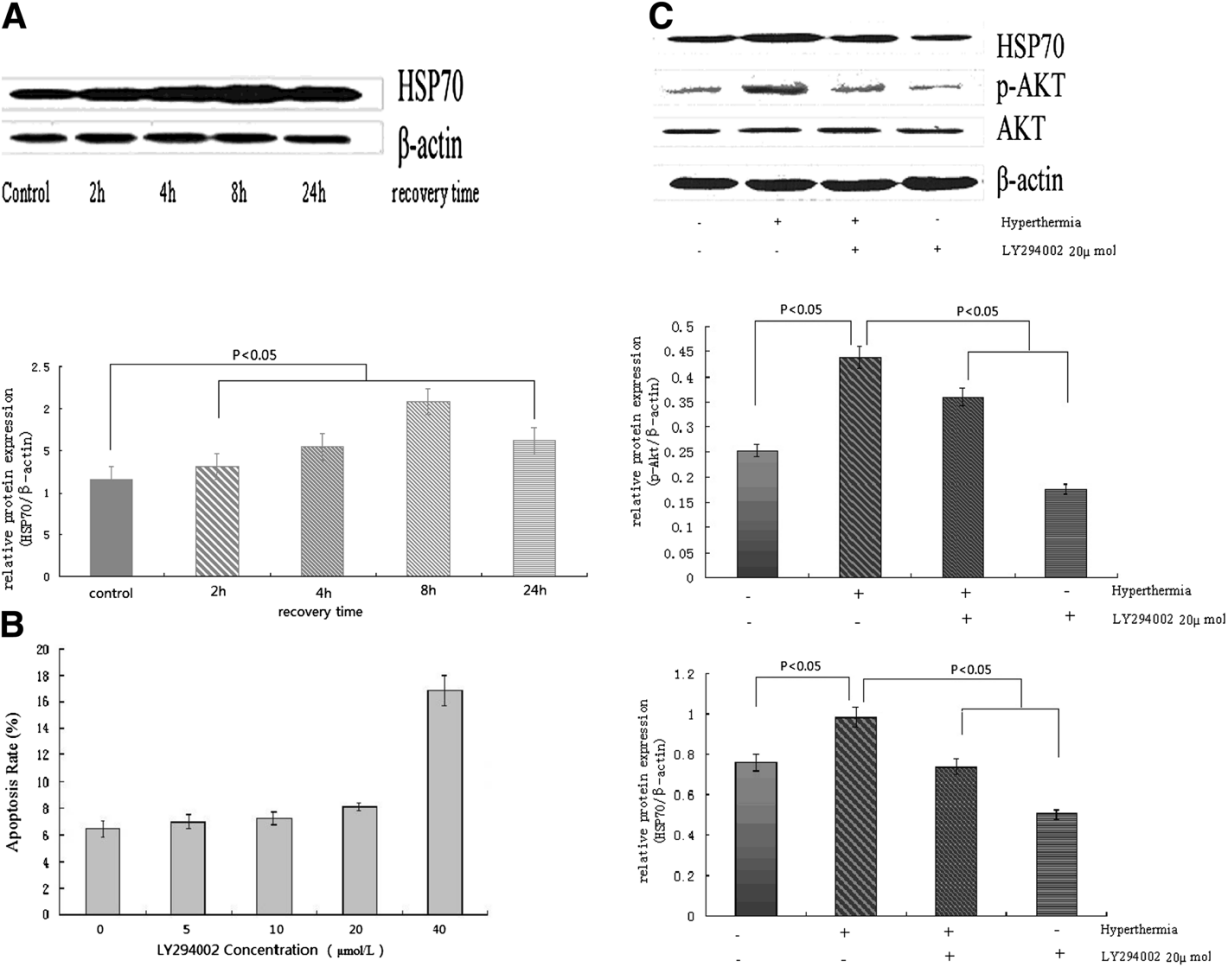
**Effects of HT and LY294002 on expression of HSP70 and cell apoptosis.** (**A**) Raji cells were cultured at 42.0°C for 1 hour and then they were returned to 37.0°C for the indicated time of recovery. The expression of HSP70 was detected by Western-Blot. (**B**) Apoptosis rate of Raji cells induced by LY294002 at indicated concentration was measured by FACS. (**C**) Raji cells were cultured at 42.0°C for 1 hour and then they were returned to 37.0°C for 8 hours or received LY294002 (20 μM) teratment for 8 hours. Expression of HSP70 and p-AKT in Raji cells were measured by Western-Blot.

Raji cells were used for the present study. We found that the apoptosis rate of cells in HT treated cells was similar to that in cells without HT treatment (data was not shown). However, HSP70 expression was enhanced obviously by exposure of HT and increased in a time-dependent manner in the first 8 hours. After 24 hours, the expression of HSP70 was still significantly higher than untreated controls (P < 0.05) (Figure 1A).

LY294002, a PI3K inhibitor, was used to block PI3K/AKT pathway. In order to explore the apoptosis-inducing effect of LY294002 on Raji cells, we detected the apoptosis rate of Raji cells after treatment with LY294002. As shown in Figure 1B, there was no difference in control group and LY294002 group when its concerntration was at 5 μM, 10 μM, and 20 μM. However, LY294002 at 40 μM could increase apoptosis rate obviously. In our following experiment, we used 20 μM of LY294002 to analyze its effect on expression of HSP70 and p-AKT in Raji cells. We found that HT could significantly upregulate the expression of HSP70 and p-AKT expression obviously while LY294002 (20 μM) could inhibit their expression dramatically (P < 0.05) (Figure 1C). These results showed that expression of HSP70 and activation of AKT (p-AKT) were attenuated significantly by LY294002 at the concentrations of 20 μM.

### DDP induced apoptosis was protected by HT and promoted by LY294002

**Figure 2:**
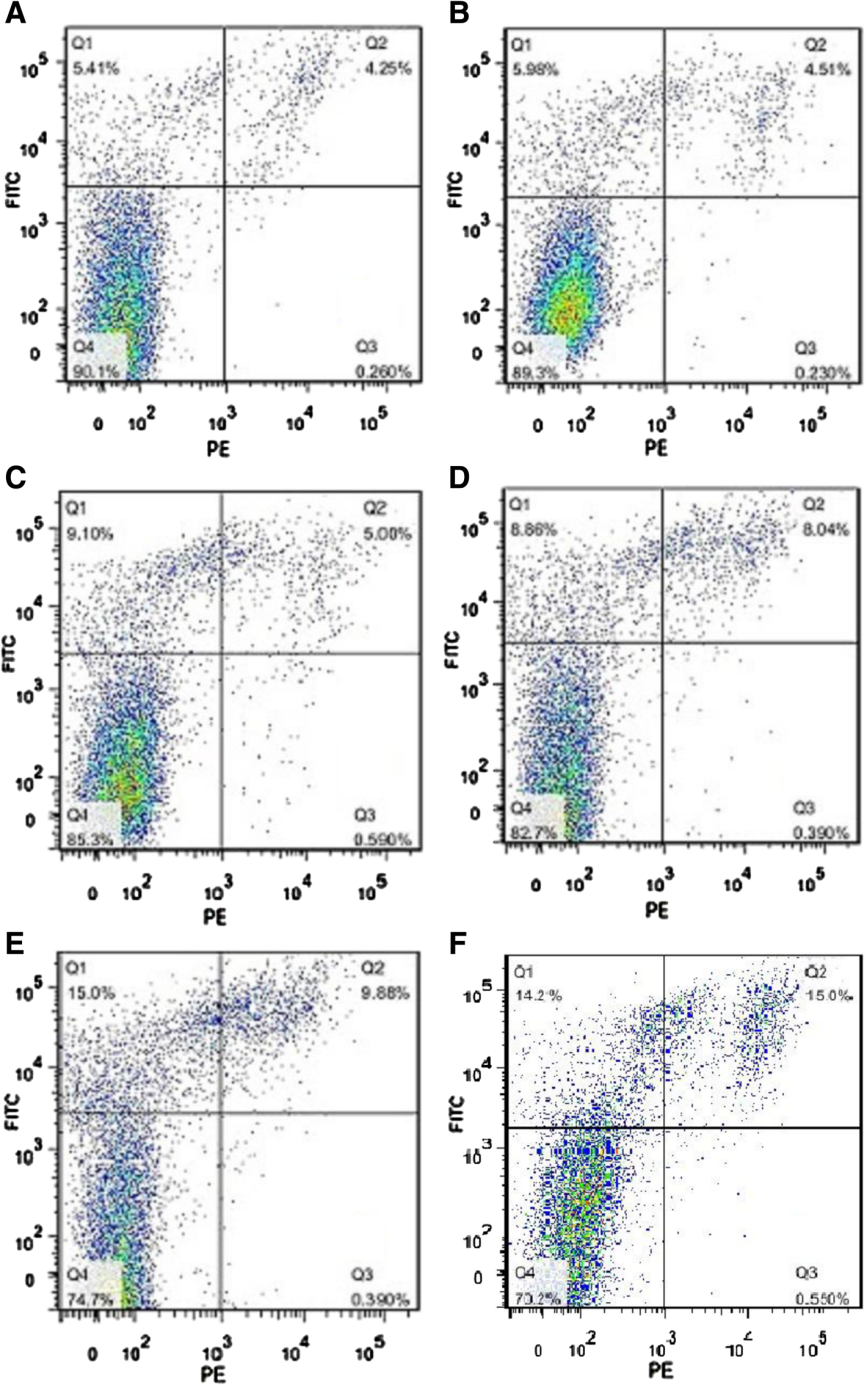
**Apoptosis induced by HT, LY294002 and DDP.** (**A**) Apoptosis of Raji cells in HT + DDP(5 μg/ml) group; (**B**) Apoptosis of Raji cells in HT + DDP(10 μg/ml)group; (**C**) Apoptosis of Raji cells in DDP(5 μg/ml)group; (**D**) Apoptosis of Raji cells in DDP(10 μg/ml)group; (**E**) Apoptosis of Raji cells in LY294002 + DDP(5 μg/ml)group; (**F**) Apoptosis of Raji cells in LY294002 + DDP(10 μg/ml)group.

To determine whether PI3K/AKT pathway and HSP70 protein expression were involved in the cancer cell sensitivity to chemotherapy, cells were incubated with LY294002 (20 μM) or HT treatment (42.0°C for 1 hour) before they were exposured to chemicals. As shown in Figure 1A, 42.0°C was enough to induce the expression of HSP70. In addition, 20 μM of LY294002 could inhibit HSP70 expression dramatically and has no effect on Raji cells apoptosis. DDP at different concentrations was added into the medium of Raji cells, HT treated cells and LY294002 treated cells. After incubated with DDP for 8 hours, cells were collected and cell apoptosis index was analyzed by flow cytometery (Figure 2). We showed that cell apoptosis was induced by DDP in a concentration-dependent manner in DDP group (14.1 ± 0.5% at 5 μg/ml, 16.9 ± 1.14% at 10 μg/ml, P < 0.01) (Figure 2A, B), while the apoptotic cells in HT + DDP group was not increased with the increasing of DDP concentration (9.73 ± 0.28% at 5 μg/ml, 10.50 ± 0.85% at 10 μg/ml, P > 0.05) (Figure 2C, D). In LY294002 + DDP treated group, the apoptosis rates of Raji cells were 25.2 ± 0.42% and 31.5 ± 0.37% respectively when the concentration of DDP were 5 μg/ml and 10 μg/ml (Figure 2E, F). Furthermore, when DDP concentration was equal, the apoptosis rate in LY294002 + DDP group was significantly higher than DDP group and the percentage of apoptotic cells in HT + DDP group was significantly lower than DDP group (P < 0.05). These results indicated that HT contributed to chemotheapeutic tolerance of Raji cells, while LY294002 promoted DDP induced cell apoptosis.

### HT protected Raji cells from chemotherapy while LY294002 enhanced their sensitivity to chemotherapy

**Figure 3:**
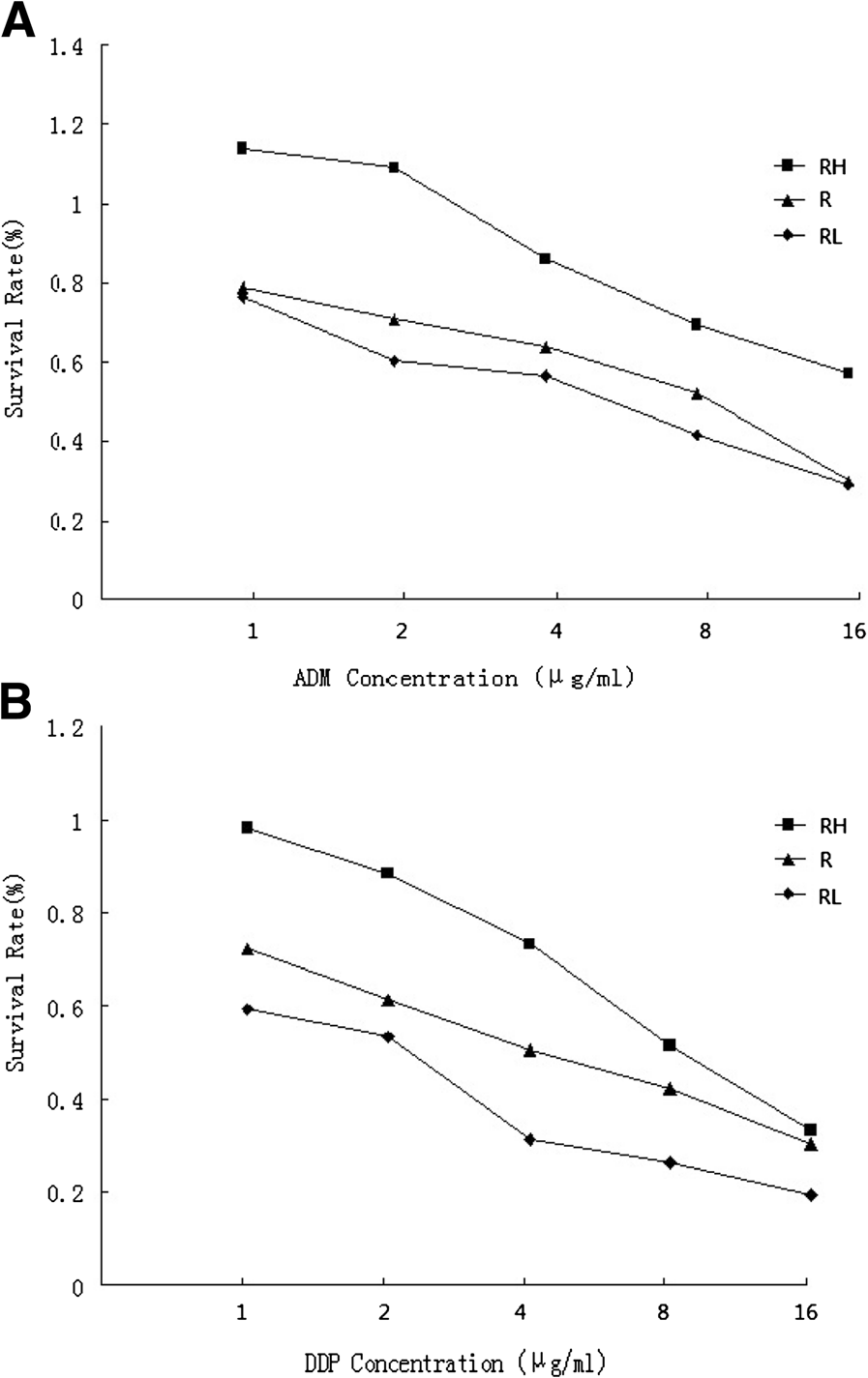
**Effects of HT and LY294002 on ADM and DDP induced cytotoxicity.** WST-8 assay was used to detect survival rates of Raji cells in indicated groups. Cell viability were inhibited by ADM in a concentration-dependent manner. Raji cells in HT + ADM group (RH gropup) showed more resistant to ADM than ADM group (R group). When treated with LY294002 and ADM (RL group), Raji cells were more sensitive to ADM (**A**). Similar phenomenon was also observed when Raji cells were treated with DDP (**B**).

Besides the effects of HT and LY294002 on cell apoptosis, other mechanisms in cell growth caused by HT and LY294002 were also explored. We next examined cell survival by WST-8 assay. As shown in Figure 3A, cell viability were inhibited by ADM in a concentration-dependent manner. Raji cells in HT + ADM group showed more resistant to ADM than ADM group and IC50 increased from 4.5 μg/ml to 15.9 μg/ml. When treated with LY294002 and ADM, Raji cells were more sensitive to ADM and the IC50 was 3.6 μg/ml. Similar phenomenon was also observed when Raji cells were treated with DDP. The IC50 in DDP group, HT + DDP group and LY294002 + DDP group were 5.2 μg/ml, 10.1 μg/ml and 2.6 μg/ml respectively (P < 0.05) (Figure 3B).

## Discussion

In this study, we observed that hyperthermia activated the expression of HSP70 in Raji cells. High expression of HSP70 was associated with lower apoptosis rate and resistance to chemotherapy drugs. Blockade of PI3K/AKT pathway not only inhibited HSP70 expression, but also contributed to chemotherapy sensitivity of Raji cells. Our findings suggested targeting PI3K/AKT/HSP70 pathway may decrease chemotherapy drugs dosage and overcome drugs resistance in Raji cells.

HSP70, one of the most important members of HSPs family, its up-regulation is known to occur as a part of the cellular defense system against environmental stresses such as heat shock, oxidative stress, or treatments with chemical agents. Previous studies indicated that Hsp70 played an important role in the control of cell cycling and growth. Up-regulation of HSP70 also has been shown to contribute to cancer cell survival via multiple anti-apoptotic functions [17]. HSP70 inhibits p53 [18] at the premitochondrial level [19], prevents mitochondrial outer membrane permeabilization (MOMP) by blocking BAX translocation and inhibit the release of cytochrome at the mitochondrial level [20,21], blocks the recruitment of procaspases 9 and 3 to the apoptosome complex, and the assembly of functional apoptosome at the postmitochondrial stage [22]. It also inhibits the activation of AIF and JNK, which induce apoptosis in a caspases-independent way [23,24]. These findings indicated a distinct and extensive anti-apoptotic function of HSP70.

Accumulating evidences indicated that hyperthermia induced HSP70 expression was related with chemotherapy resistance [25]. Moreover, Behnsawy et al had proved that the expression of HSP70 was correlated with cell survival when treated with chemotherapy [26]. In acute myeloid leukemia and acute lymphoblastic leukemia, HSP70 is an indicator for poor prognosis [27]. However, the precise mechanism of Hsp70 in lymphoma cell survival and chemotherapy resistance remains unclear. In the study, we demonstrated that hyperthermia induced HSP70 expression and protected Raji cells against ADM and DDP treatment.

The induction of HSP70 in response to various stimuli is mainly regulated by the activation of HSF1 [28]. Recent studies have showed that PI3K/AKT signal pathway was involved in the induction of HSP70 expression [29,30]. This regulation might rely on the fact that the activation of AKT inhibited GSK-3β [31], and the inhibition of GSK-3β could activate HSF1. In our study, we blocked PI3K/AKT signaling pathway by LY294002 and detected the altered expression of HSP70 in Raji cells. Our results indicated the blockade of PI3K/AKT signaling pathway inhibited HSP70 expression obviously. However, some studies demonstrated that these signal cascades were not found in either lens endothelial cells nor U937 cells under certain conditions [32,33]. These contradictory phenomenons might be incriminated to cell heterogeneity as well as different disposal conditions.

PI3K/Akt signaling pathway is frequently activated in malignant diseases, including many hematologic malignancies [34] and contributes to tumor cell survival [35-38]. The up-regulation of this signal pathway was also suggested to increase drug resistance [39], but the underlying mechanism is not fully proved. In this study, we confirmed the existence of PI3K/AKT/HSP70 pathway in Raji cells. Blockade of PI3K/AKT pathway not only inhibit HSP70 expression, but also promote cell sensitivity to chemotherapy. Given the relationship between HSP70 expression and chemotherapy resistance, we concluded that down-regulation of HSP70 was at least partly responsible for the promotion of Raji cells sensitivity to chemotherapy. Inhibition of HSP70 may be an encouraging method to overcome chemotherapy resistance.

Antisense HSP70 oligomer and HSP70 siRNA have been used to induce low expression of HSP70 in different kinds of cells and resulted higher apoptosis rate [40,41]. However, there are still some problems in their clinical application. Quercetin, a kind of flavanoid, inhibited HSPs expression in many kinds of cancer cells [42,43]. Cells became more susceptible to apoptosis when treated with quercetin followed by hyperthermia, chemotherapy or radiotherapy [44-46]. But quercetin showed significant toxicity even used as a signal agent, which largely limited its clinical application. HSP90, another important member of HSPs family, is closely related with cancer treatment. One of its specific inhibitor, 17-allylamino-demethoxy-geldanamycin (17-AAG), had been presented in the completed stage I clinical trial and demonstrated a favorable therapeutic effect. Another inhibitor PU-H71 has demonstrated considerable anti-tumor activity in diffused large B cell lymphoma and multiple myeloma [47]. So far there is no specific small-molecule HSP70 inhibitor which is available comparable with 17-AAG.

In summary, we documented that PI3K/AKT/HSP70 pathway existed in Raji cells and revealed an intriguing mechanism that down-regulation of HSP70 was responsible for the promotion of sensitivity to chemotherapy in Raji cells. Targeting PI3K/AKT pathway or inhibiting HSP70 expression may be an encouraging method to overcome chemotherapeutic resistance.

## Competing interest

The authors have no relevant conflicts of interest.

## Authors’ contributions

XW designed research. XF, YJ and LF performed research. HC was responsible for statistical analysis. CZ drafted the manuscript. MD revised it critically for important intellectual content. All authors read and approved the final manuscript.
